# Supplementary material for: Learning curve and surgical outcome of robotic assisted colorectal surgery with ERAS program
Source: Sci Rep. 2022 Nov 29;12:20566. doi: 10.1038/s41598-022-24665-w (PMC9709162; doi:10.1038/s41598-022-24665-w)
Supplement: Supplementary file 1 — Supplementary Information 1. [file 41598_2022_24665_MOESM1_ESM.zip › LSC Raw data-Ñ[▒K/109/P_ñw╝╨░O▒Kñσ.pdf]

[redacted]

Pathologic diagnosis:
1. Sigmoid colon, laparoscopic anterior resection --- Status post polypectomy with focal mild xanthomatous inflammation. No residual tumor seen. The bilateral cut ends and mesocolic lymph nodes (0/21) are free of tumor.
2. Colon, cut end, distal, resection --- Chronic inflammation and mild mucosal hyperplasia.
3. IMA root, dissection --- Unremarkable fibroadipose tissue and lymph node (0/1).

Prognostic and predictive factor:
(Combined with polypectomy specimen: S0937414)
1. Tumor size: 0.6 cm (measured on slides of S0937414).
2. Depth of invasion: Submucosa.
3. Histologic grade: G2: Moderately differentiated.
4. Angiolymphatic invasion: Not identified.
5. Perineural invasion: Not identified.
6. Tumor deposits (discontinuous extramural extension): Not identified.
7. Macroscopic tumor perforation: Not identified.
8. Surgical Margins: a) Proximal Margin: Uninvolved, distance of stitch site from margin: 9.5 cm. b) Distal Margin: Uninvolved, distance of stitch site from margin: 6 cm. c) Radial or Mesenteric Margin: Uninvolved.
9. Lymph node status: Uninvolved (0/22).
10. Extranodal involvement: Not identified.
11. Tumor regression grading S/P CCRT: No known presurgical therapy.
12. Type of polyp in which invasive carcinoma arose: Tubulovillous adenoma. (S0937414)
13. Additional pathologic findings: Not identified.
14. Pathological TNM Stage: pT1N0(According to the eighth edition, American Joint Committee on Cancer Staging Guidelines for Tumors).
15. TNM descriptors: Not applicable.

Gross description:
The specimen consists of 1) a segment of colon, 15 cm in length and 5 cm in bilateral cut ends circumference, and mesocolic soft tissue. There is a surgical tie labeling previous polypectomy site, which is 6 cm to the nearest cut end. There is no obvious scar or tumor seen at mucosa. The both cut ends are free of tumor grossly. 2) distal cut end, a piece of tan mucosa-covered soft tissue, 1.6x1.5x1 cm. 2) IMA root, a piece of tan yellow soft tissue, 2.8x2.5x0.8 cm.
Representative part for section: A) proximal cut end B) distal cut end C-D) stitch site E) radial margin H-) lymph nodes F) specimen 2 G) specimen 3. Blue ink indicated serosa; yellow ink indicated radial margin; green ink indicated stitch site.
#T-59470_2 #M-43000_2 0 1105 SP0000
#T-C4560_2 #M-09183_2 0 1105 000000

已電子簽章

敬告：本單僅供醫師參考使用，不具任何申請效力！！
請病患妥為保管，若不慎遺失需自負個人資料外洩責任！
